# The Influence of Selected Plant Growth Regulators and Carbohydrates on In Vitro Shoot Multiplication and Bulbing of the Tulip (*Tulipa* L.)

**DOI:** 10.3390/plants12051134

**Published:** 2023-03-02

**Authors:** Dariusz Sochacki, Przemysław Marciniak, Maria Ciesielska, Janina Zaród, Sutrisno

**Affiliations:** Section of Ornamental Plants, Warsaw University of Life Sciences, 166 Nowoursynowska Street, 02-787 Warsaw, Poland

**Keywords:** ‘Heart of Warsaw’, meta-topolin, micropropagation, paclobutrazol, ‘Serce Warszawy’

## Abstract

The aim of this study was to check the effects of sugar type on the in vitro shoot multiplication of the tulip cultivar ‘Heart of Warsaw’ and the effects of paclobutrazol (PBZ) and 1-naphthylacetic acid (NAA) on the bulbing of previously multiplied shoots. In addition, the subsequent effects of previously used sugars on the in vitro bulb formation of this cultivar were checked. First, the optimum supplementation of Murashige and Skoog medium with plant growth regulators (PGRs) was selected for shoot multiplication. Of the six tested, the best results were obtained using a combination of 2iP 0.1 mg·L^−1^, NAA 0.1 mg·L^−1^, and mT 5.0 mg·L^−1^. The effects of different carbohydrates (sucrose, glucose, and fructose at 30 g·L^−1^ and a mixture of glucose and fructose at 15 g·L^−1^ each) on multiplication efficiency was then tested on this medium. The microbulb-forming experiment was carried out taking into consideration the effects of previously applied sugars, and at week 6, the agar medium was flooded with liquid medium containing NAA 2 mg·L^−1^, PBZ 1 mg·L^−1^, or medium without PGRs; in the first combination, the cultures were left on a single-phase medium, solidified with agar, as a control. After 2 months of treatment at 5 °C, the total number of microbulbs formed and the number and weights of mature microbulbs were assessed. The results obtained indicate the ability of using meta-topolin (mT) in tulip micropropagation and point to sucrose and glucose as the optimal carbohydrates for intensive shoot multiplication. The results lead to the conclusion that it is most advantageous to multiply tulip shoots on glucose medium and then to carry out cultures on a two-phase medium with PBZ, which results in a higher number of microbulbs and their faster maturation.

## 1. Introduction

The genus *Tulipa* L. (family *Liliaceae*) belongs to the monocotyledonous plants whose storage organ is a bulb usually made up of four or five scales [1,2], named also as the geophyte. This genus includes 80 to 105 species found in the wild of North Africa, Southern Europe, and Central Asia. Multiple wild species and uncontrolled outcrossing have made classifications within this genus difficult and frequently changing [3,4,5,6]. Based on the diversity of their flower shape and size, they have been divided into 15 horticultural classification groups [7], but as of 2018, a 16th group—Crown Tulips—has emerged [8].

The tulip is one of the world’s most popular ornamental geophytes with a wide range of uses. It is utilized as a cut and potted flower (mainly from forcing), as a garden plant, and in seasonal and perennial plantings in urban green spaces. An area of nearly 10,000 ha is dedicated for tulip bulb production in the Netherlands, the leading producer of flowers. In terms of sales value, the tulip has been in the top three cut flowers sold in the Netherlands for many years, after roses and chrysanthemums [9].

As societies develop economically and become more prosperous, the demand for flowers and bulbs has increased. This has resulted in the continuous creation of new cultivars, as well as the search for more effective, efficient methods of propagation and production of flower bulbs. Tulips can be propagated by seeds (for breeding purposes only), separating adventitious bulbs, and by micropropagation [5,10]. Micropropagation makes it possible, on appropriately selected media, to increase productivity; however, in the case of the tulip, shortening the propagation period with this method is debatable because obtaining the storage organs, i.e., microbulbs, is much more difficult and, takes more time compared to rooted shoots of nonbulbous plants. Direct organogenesis [11,12,13,14,15] and callusogenesis including somatic embryogenesis [16,17,18] are in vitro techniques for tulip reproduction.

Cyclic shoot multiplication on the Murashige and Skoog (MS) [19] medium in the presence of thidiazuron (TDZ), N^6^-(-isopentyl)adenine (2iP), and 1-naphthaleneacetic acid (NAA) has shown great promise and has been used for tulips propagation on a quite massive scale. From one bulb, 500 to 2000 microbulbs (bulblets) can be obtained in 2–3 years [20,21]. It has also contributed to increasing virus-free material and inducing mitotic tetraploids [22,23].

Although micropropagation has advantages, it is worth remembering that sometimes mutations can occur and genetically identical plants cannot be obtained. Changes in the quantity or quality of DNA can also be caused by other factors, such as the selection of growth regulators. A concentration of cytokinins that is too high accompanied by insufficient amounts of auxins can cause abnormalities in DNA material [15]. In addition, the time of the culture can also have an impact [24]. For example, with the cyclic shoot multiplication method, it is not recommended to maintain such a culture for too long due to the occurrence of unfavorable somaclonal changes [25].

Over the years, knowledge of the action of plant growth regulators (PGRs) has significantly deepened, which has allowed for a more precise understanding of their functioning and influence on plant metabolism. To date, the role of many plant-growth-regulating compounds with respect to the regulation of plant growth processes, seed germination, and rooting as well as their effects on biochemical changes in plants are well understood activities [26,27]. However, for newly introduced compounds, such as meta-topolin (mT), there is little or no information, particularly in the case of tulip micropropagation. PGRs are the substances that, even in small amounts, affect metabolism and physiological processes in plants [28]. They are chemical messengers that control cell activity and their effects can be detected at sites distant from the site of biosynthesis. Different groups of growth regulators can act synergistically and antagonistically towards each other [26].

In experiments conducted on tulips, it has been found that the type of sugar used influences shoot multiplication and also the subsequent efficiency of in vitro bulb formation [29]. The most widely used carbohydrate in tissue culture is sucrose; the results of using sucrose are often very favorable or no worse than other sugars [30]. According to Custers et al. [31], a medium (MS) consisting of 4% sucrose, 500 mg·L^−1^ tryptone, and 4 nM 1-naphthaleneacetic acid (NAA), with 0.75% agar was instrumental in saving embryos of the interspecific hybrid *T. gesneriana* × *T. kaufmanniana*. In a study by Van de Wiel et al. [32], the authors increased the sucrose concentration to 9% and also reported a positive effect on embryo survival. It is well known that simple carbohydrates such as glucose or fructose should be very easily taken up by plant cells [33]. However, there are few reports on their use in geophyte propagation in tissue culture.

The aim of this study was to investigate the possibility of using aromatic cytokinin mT for micropropagation of the tulip cultivar ‘Heart of Warsaw’, the effects of sugar types on the in vitro shoot multiplication, and the effects of PBZ and NAA on the bulbing of previously multiplied shoots. In addition, the subsequent effects of previously used sugars on the in vitro bulb formation of this cultivar was checked.

## 2. Results and Discussion

### 2.1. Experiment 1

Effects of selected plant growth regulators (PGRs) on in vitro shoot multiplication of the tulip cultivar ‘Heart of Warsaw’

Effects of PGRs used in the MS medium on the shoot multiplication of the tulip cultivar ‘Heart of Warsaw’ were observed (Table 1).

The highest number of shoots per cluster (9.14) was obtained on the MS medium supplemented by using 2iP 0.1 mg·L^−1^, NAA 0.1 mg·L^−1^, and mT 5 mg·L^−1^. The lowest number of shoots (3.32) was achieved on the MS medium supplemented by using 2iP 0.1 mg·L^−1^, IBA 0.1 mg·L^−1^, and mT 5 mg·L^−1^. It is worth noting that, in this case, only replacing the auxin IBA with NAA resulted in an almost three-fold increase in the number of shoots obtained. The number of shoots obtained on the MS medium with TDZ 0.1 mg·L^−1^ and NAA was lower than those on the MS media with mT 0.1 mg·L^−1^ and with some auxin NAA.

The obtained results concerning the effects of selected PGRs added to the MS medium on the in vitro shoot multiplication of the tulip cultivar ‘Heart of Warsaw’ indicate that the optimal medium is a medium enriched with 2iP 0.1 mg·L^−1^ and mT 5 mg·L^−1^ in combination with auxin NAA 0.1 mg·L^−1^. Given that even a small change in the composition of the MS medium in terms of the type of auxin used (replacing IBA for NAA) resulted in extremely different results, this may be confirmation of the importance of selecting the optimal combination of PGRs for each cultivar. This may be linked to the composition of endogenous growth regulators [34]. In addition, for the tulips ‘Recreado’ and ‘Christmas Marvel’, NAA has been proven to be the auxin with the best effect on shoot proliferation, but only when it interacted with cytokinin BA [35]. As in previous experiments, 2iP on tulip gave satisfactory results [36,37,38]. TDZ, as compared to mT, could cause stem necrosis or inefficient proliferation [39], which is why it is important to demonstrate in our own studies the possibility of effectively replacing TDZ with a new aromatic cytokinin such as mT. The effectiveness of replacing TDZ with mT cytokinin is also supported by the results shown in Table 2.

### 2.2. Experiment 2

Effects of different carbohydrates on in vitro shoot multiplication of the tulip cultivar ‘Heart of Warsaw’.

**Figure 1:**
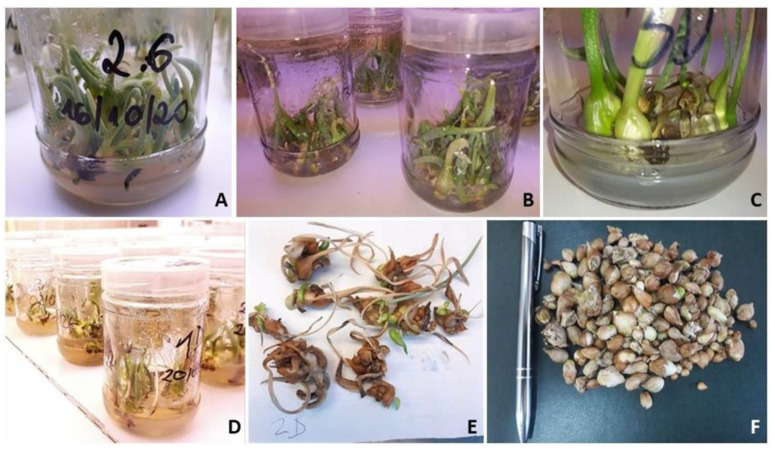
Different micropropagation stages of the tulip cultivar ‘Heart of Warsaw’: (**A**) Shoot multiplication on MS medium with glucose; (**B**) no or very limited bulb formation on the control medium without liquid phase; (**C**) start of microbulbs forming on the two-phase medium with fructose and paclobutrazol; (**D**) cultures on two-phase medium, the preparatory stage for bulbing, Experiment 3; (**E**) microbulbs obtained on medium supplemented with glucose and paclobutrazol which are mostly mature, surrounded by a dry covering scale; (**F**) matured microbulbs.

The type of carbohydrate added to the nutrient solution had a significant effect on the shoot proliferation of the tulip cultivar ‘Heart of Warsaw’, but not at every stage of multiplication (Table 3). The results obtained and confirmed by statistical analysis indicate that sucrose and glucose are the optimal sugars for intensive shoot multiplication (Figure 1A).

The addition of fructose or a mixture of fructose and glucose to the medium composition resulted in less proliferating shoots. Completely different results were obtained by Podwyszyńska [29] for the cultivars ‘Black Parrot’, ‘Lustige Witwe’, and ‘Blenda’. There, the lowest results were obtained on a medium with sucrose, while the highest results were obtained on a medium with fructose or its mixture with glucose [29].

Furthermore, it was noted that media containing fructose solidified less well than those containing other sugars. This may be due to a reduction in the pH of the medium after autoclaving. After sterilization, a drop in pH can be observed from pH = 5.8 to even below pH = 4.5. At this low pH, growth regulators are degraded, and also the agar does not solidify the media effectively [40]. However, this does not justify obtaining proper solid media for other sugars. There are no data in the literature on this issue.

Although the negative effect of fructose on shoot multiplication may be justified by the toxic nature of its hydrolysis products after autoclaving [41], in general, the above information confirms the need for individual studies for each tulip cultivar in terms of both PGRs and the choice of carbohydrates used.

### 2.3. Experiment 3

Effects of sugar type and NAA and paclobutrazol (PBZ) on in vitro bulbing of the tulip cultivar ‘Heart of Warsaw’.

Our experiments showed the effects of PGRs used on the formation of tulip microbulbs in vitro (Table 4 and Table 5) (Figure 1B–D). A higher total number of bulbs produced was observed on the two-phase medium containing PBZ than on both control media (Table 4). The addition of NAA to the liquid phase of the medium resulted in a similar total number of microbulbs (matured and immatured) as the medium with the addition of PBZ, but a statistically significant higher number of mature bulbs was observed on the latter medium. The number of fully mature bulbs was higher on the two-phase medium containing PBZ than on both control media and the liquid medium with NAA (Table 5). This indicates that PBZ, which is a growth retardant, definitely accelerated the bulb maturation process. This substance is known as an antagonist of gibberellin (GA) and inhibitor of GA biosynthesis. There are many examples of how a reduction in GA synthesis during in vitro storage organ formation stimulates the formation of bulbs, corms, and tubers of many ornamental geophytes [42]. Of the many growth retardants, PBZ has shown high efficacy in this respect for *Gladiolus* L. [43], *Lilium* L. ‘Star Gazer’ [44], *Lilium* L. ‘Starfighter’ [45], *Lilium monadelphum* M. Bieb. var. *armenum* [46], *Hippeastrum hybridum* Hort. [47], *Gloriosa rothschildiana* O’Brien [48], and *Leucojum aestivum* L. [49]. In tulips (*Tulipa* L.), PBZ stimulated direct bulb regeneration on initial explants [14] and bulb formation in the system based on cyclic multiplication of adventitious shoots [50].

A subsequent effects of the types of sugar added to the medium at an earlier stage of the experiment on microbulb formation are also shown (Table 4 and Table 5), but only for the total number of bulbs (mature and immature). A higher total number of bulbs was obtained on the medium in which glucose was used. The combination containing a mixture of glucose and fructose also performed comparably to it in absolute terms, but the number of microbulbs obtained was not statistically different. Fewer bulbs were produced by shoots that had previously grown on sucrose or fructose media. For both the number of mature microbulbs and the weight of mature microbulbs (Figure 1F), there were no subsequent effects of the type of sugar added to the medium (Table 6).

As a conclusion, the results obtained indicate that the method for obtaining tulip microbulbs by cyclic shoot multiplication can be further improved. The possibility of using mT in tulip micropropagation, previously not used in the in vitro cultures of this species, has been demonstrated. The results also confirm the need to test and specify the procedure for specific tulip cultivars, as each may respond slightly differently to the composition of the medium and in vitro culture conditions.

## 3. Materials and Methods

### 3.1. Plant Material

In the experiments, we used material from stabilized in vitro cultures of the tulip cultivar ‘Heart of Warsaw’ (*Tulipa* L.). This new cultivar functions under two equivalent names: the Polish name ‘Serce Warszawy’ and the English name ‘Heart of Warsaw’. The cultivar has been entered in the International Register of Tulip Cultivars kept in the Netherlands by the Royal General Flower Bulb Association in Hillegom (De Koninklijke Algemeene Vereeniging voor Bloembollencultuur, KAVB), under its English name [51]. It is a tall, late-flowering cultivar, bred in Poland by Roman Szymański, with dark red petals and a yellow pistil stigma, from the Fringed tulip group. The cultivar is in the ownership of the Royal Castle in Warsaw, and its flowers have decorated the castle gardens since 2019 (Supplementary Materials).

### 3.2. Terms and Conditions for Conducting Cultures

The in vitro cultures were conducted in a phytotron, with a 16 h photoperiod and illumination with fluorescent white light (25 μM m^−2^·s^−1^ intensity), at 22 ± 1 °C. The pH of the media was adjusted to 5.6 with NaOH before autoclaving at 121 °C and an overpressure of 1.2 kg/cm^2^ (110 kPa) for 20 min.

#### 3.2.1. Experiment 1

Effects of selected plant growth regulators (PGRs) on in vitro shoot multiplication of the tulip cultivar ‘Heart of Warsaw’.

Several-week-old tulip shoot cultures of ‘Heart of Warsaw’ initiated earlier from flower stems were transferred to Murashige and Skoog [19] medium (MS) supplemented with vitamins, inositol 0.1 g·L^−1^, casein hydrolysate 1 g·L^−1^, adenine sulfate 40 mg·L^−1^, and sucrose 30 g·L^−1^, and all were solidified with agar (Difco Bacto Agar) 8 g·L^−1^, with several modifications in meaning of PGRs (Table 7).

Each 180 mL jar contained approximately 20 mL of medium and four clumps on which shoots developed. Each jar represented a separate replicate. Every 6 weeks, the clumps were transferred to fresh medium. They were also divided with a scalpel so that there were 3 shoots on each clump. After 20 weeks (after 3 passages), the results were collected. The number of shoots in each repetition for each starter clump was counted. The counted shoots had to be greater than or equal to 5 mm in length.

A similar experiment (as the second repetition) using three modifications of the MS medium used in the first repetition (1, 3, and 5 listed in the Table 1) was performed.

#### 3.2.2. Experiment 2

Effects of carbohydrate type on in vitro shoot multiplication of the tulip cultivar ‘Heart of Warsaw’.

The shoot clumps of the tulip cultivar ‘Heart of Warsaw’ were used as experimental material. The clumps were appropriately divided so that there were only a few shoots on each clump. Then, the prepared material was placed in jars (300 mL in volume) with about 20 mL of nutrient solution comprosed of MS medium supplemented with vitamins, inositol 0.1 mg·L^−1^, casein hydrolysate 1 mg·L^−1^, adenine sulfate 40 mg·L^−1^; PGRs (selected according to their positive effects on shoot proliferation in Experiment 1) 2iP 0.1 mg·L^−1^, NAA 0.1 mg·L^−1^, and mT 5 mg·L^−1^; and agar (Difco Bacto Agar) 9 mg·L^−1^, with several modifications regarding the type and content of carbohydrates (Table 8).

In each jar with medium, 4 clumps with 3–4 shoots were placed. Each jar constituted a separate replicate. The clumps were passaged every 6 weeks with fresh medium. They were also divided with a scalpel so that there were 3 shoots on each clump. The number of shoots in each repetition for one starter clump was counted after the 1st, 2nd, 3rd, and 4th passages. The counted shoots had to been greater than or equal to 5 mm in length. The multiplication rate, i.e., the increase in the number of shoots over successive passages, as well the total multiplication rate after the 4th passage were assessed. The number of shoots obtained after the 4th passage per one starting clump was also assessed.

#### 3.2.3. Experiment 3

Effects of sugar type and NAA and paclobutrazol on in vitro bulbing of the tulip cultivar ‘Heart of Warsaw’.

In the first stage of the experiment, healthy clumps containing 3–4 shoots were selected from the cultures of Experiment 2 and placed, three per jar, on media analogous to that of Experiment 2. There were 18 jars in each treatment, and each jar was an experimental repetition. In the second stage, for further culture, a two-phase medium was used: solid and liquid. After 6 weeks, the shoot cultures were flooded with MS liquid medium (without agar) in a volume of about 80% of the volume of solid medium, containing 30 g·L^−1^ sucrose in each treatment and differing in the content of PGRs:

A. First control—no liquid medium;

B. Second control—flooded with medium without PGRs;

C. Flooded with liquid medium containing 2 mg·L^−1^ NAA;

D. Flooded with liquid medium containing 1 mg·L^−1^ paclobutrazol (PBZ).

As two factors (four sugar types—1–4 and four type of medium/PGRs—A–D) were included in the experiment, sixteen experimental treatments were created (1A, 1B, 1C, 1D, 2A, 2B, 2C, 2D, 3A, 3B, 3C, 3D, 4A, 4B, 4C, 4D).

In the third stage, after another 6 weeks, all cultures were replanted to MS solid medium without PGRs, containing 60 g·L^−1^ sucrose. The jars were then placed in the refrigerator, at 5 °C, in darkness for 2 months.

After two months, the plants were transferred to standard MS medium and placed in a phytotron under 16 h photoperiod conditions. After 3 months, the total number of bulbs produced, the number of mature bulbs, and the weights of mature bulbs were assessed. Bulbs with dry scales and capable of surviving in ex vitro conditions were considered mature. The results obtained were statistically analyzed by one-way (Experiments 1 and 2) or two-way (Experiment 3) analysis of variance (ANOVA) using the IBM SPSS Statistics PS Imago Pro software, and the means were compared by using Duncan’s test at a significance level of *p* = 0.05.

## 4. Conclusions

1. For the tulip cultivar ‘Heart of Warsaw’, the most effective composition of PGRs which contributed to the highest number of shoots was the addition to the MS medium of 2iP 0.1 mg·L^−1^, NAA 0.1 mg·L^−1^ and mT 5 mg·L^−1^. The results obtained indicate the possibility of using mT in tulip micropropagation.

2. The type of carbohydrate added to the nutrient solution had a significant effect on the shoot proliferation of the tulip cultivar ‘Heart of Warsaw’. The results obtained indicate that sucrose and glucose were the optimal sugars for intensive shoot multiplication.

3. The conjunction of propagating shoots of the tulip cultivar ‘Heart of Warsaw’ on glucose medium and continuing cultures on two-phase medium with PBZ leads to a higher number of microbulbs and their faster maturation.

## Figures and Tables

**Table 1:** Effects of selected plant growth regulators (PGRs) on in vitro shoot multiplication of the tulip cultivar ‘Heart of Warsaw’.

PGRs Added to MS Medium	Number of Shoots per Clump ± SD
TDZ 0.1 mg·L^−1^ + NAA 0.1 mg·L^−1^ + 2iP 5 mg·L^−1^	4.53 ± 1.39 ab *
TDZ 0.1 mg·L^−1^ + IBA 0.1 mg·L^−1^ + 2iP 5 mg·L^−1^	6.25 ± 2.36 bc
mT 0.1 mg·L^−1^ + NAA 0.1 mg·L^−1^ + 2iP 5 mg·L^−1^	8.54 ± 3.05 cd
mT 0.1 mg·L^−1^ + IBA 0.1 mg·L^−1^ + 2iP 5 mg·L^−1^	6.82 ± 2.82 bcd
2iP 0.1 mg·L^−1^ + NAA 0.1 mg·L^−1^ + mT 5 mg·L^−1^	9.14 ± 4.60 d
2iP 0.1 mg·L^−1^ + IBA 0.1 mg·L^−1^ + mT 5 mg·L^−1^	3.32 ± 0.96 a

* Means ± standard deviation (SD) in a column followed by the same letter does not differ significantly at α = 0.05 (Duncan test). N = 10 vessels in each treatment.

**Table 2:** Effects of selected plant growth regulators (PGRs) on in vitro shoot multiplication of the tulip cultivar ‘Heart of Warsaw’—second repetition.

PGRs Added to MS Medium (mg·L^−1^)	Multiplication Rate after 1 Passage ± SD	Multiplication Rate after 2 Passages ± SD	Multiplication Rate after 3 Passages ± SD	Total Multiplication Rate ± SD
TDZ 0.1 + NAA 0.1 + 2iP 5	1.22 ± 0.30 a *	1.10 ± 0.20 a	1.19 ± 0.22 a	1.64 ± 0.85 a
mT 0.1 + NAA 0.1 + 2iP 5	1.37 ± 0.41 a	1.20 ± 0.27 a	1.08 ± 0.25 a	1.86 ± 0.95 a
2iP 0.1 + NAA 0.1 + mT 5	1.42 ± 0.37 a	1.17 ± 0.32 a	1.10 ± 0.31 a	1.94 ± 1.08 a

* Means ± standard deviation in a column followed by the same letter does not differ significantly at α = 0.05 (Duncan test). N = 10 vessels in each treatment.

**Table 3:** Effects of different carbohydrates on in vitro shoot multiplication rate of the tulip cultivar ‘Heart of Warsaw’.

Carbohydrate	Multiplication Rate after 1 Passage ± SD	Multiplication Rate after 2 Passage ± SD	Multiplication Rate after 3 Passage ± SD	Multiplication Rate after 4 Passage ± SD	Total Multiplication Rate ± SD	No. of Shoots after 4th Passage per One Starting Clump ± SD
Sucrose	1.33 ± 0.39 b *	1.70 ± 0.55 a	1.20 ± 0.16 a	1.64 ± 0.54 b	3.01 ± 1.48 ab	34.33 ± 13.06 b
Glucose	1.36 ± 0.35 b	1.34 ± 0.39 a	1.30 ± 0.37 a	1.40 ± 0.28 ab	3.25 ± 1.03 b	35.88 ± 12.78 b
Fructose	1.19 ± 0.23 ab	1.31 ± 0.33 a	1.18 ± 0.18 a	1.26 ± 0.27 ab	2.37 ± 1.07 ab	18.95 ± 8.50 a
Glucose + Fructose	0.92 ± 0.18 a	1.28 ± 0.42 a	1.36 ± 0.32 a	1.11 ± 0.16 a	1.84 ± 0.73 a	19.80 ± 8.21 a

* Means ± standard deviation in a column followed by the same letter does not differ significantly at α = 0.05 (Duncan test). N = 10 vessels in each treatment at the beginning of the experiment, Ns differ from 6 to 9 at the date of evaluation, because of removing of contaminated cultures.

**Table 4:** Effects of the type of medium and plant growth regulators (PGRs) and the after effect of different carbohydrates on the total number of bulbs the tulip cultivar ‘Heart of Warsaw’ formed in vitro.

Type of Medium, PGRs Added to the Medium	Carbohydrate				Mean for Type of Medium
	Sucrose	Glucose	Fructose	Glucose + Fructose	
1st control, single-phase medium (without liquid medium)	6.25 ± 5.74 ab *	10.33 ± 6.81 ab	9.00 ± 4.24 ab	7.00 ± 2.65 ab	8.07 ± 4.76 a
2nd control, two-phase medium (liquid medium without PGRs)	8.80 ± 4.44 ab	8.25 ± 6.18 ab	3.50 ± 0.50 a	11.50 ± 3.87 ab	8.34 ± 4.83 a
Two-phase medium (liquid medium with 2 mg·L^−1^ NAA)	10.00 ± 2.00 ab	11.50 ± 5.20 ab	5.67 ± 4.73 ab	15.00 ± 5.96 b	11.20 ± 5.63 ab
Two-phase medium (liquid medium with 1 mg·L^−1^ PBZ)	12.67 ± 2.89 ab	28.00 ± 12.99 c	12.00 ± 7.16 ab	9.60 ± 4.10 ab	15.38 ± 10.38 b
Mean for carbohydrates	9.13 ± 4.44 a	14.80 ± 11.23 b	7.96 ± 5.53 a	11.18 ± 5.04 ab	x

* Means ± standard deviation in a column followed by the same letter does not differ significantly at α = 0.05 (Duncan test). N = 18 vessels in each treatment at the beginning of the experiment; N differs from 14 to 17 at the date of evaluation, because of removing of contaminated cultures.

**Table 5:** Effects of the type of medium and plant growth regulators (PGRs) and the after effect of different carbohydrates on the number of mature bulbs of the tulip cultivar ‘Heart of Warsaw’ formed in vitro.

Type of Medium, PGRs Added to the Medium	Carbohydrate				Mean for Type of Medium
	Sucrose	Glucose	Fructose	Glucose + Fructose	
1st control, single-phase medium (without liquid medium)	0 ± 0.00 a *	1.67 ± 0.58 a	1.50 ± 1.29 a	2.00 ± 2.00 a	1.21 ± 1.31 a
2nd control, two-phase medium (liquid medium without PGRs)	1.20 ± 1.30 a	1.25 ± 1.50 a	0.67 ± 1.15 a	4.25 ± 1.71 a	1.88 ± 1.93 a
Two-phase medium (liquid medium with 2 mg·L^−1^ NAA)	1.33 ± 1.15 a	1.50 ± 3.00 a	1.33 ± 0.58 a	2.80 ± 1.30 a	1.87 ± 1.77 a
Two-phase medium (liquid medium with 1 mg·L^−1^ PBZ)	4.33 ± 3.06 a	14.50 ± 15.72 b	3.00 ± 1.41 a	3.40 ± 2.07 a	6.85 ± 8.75 b
Mean for carbohydrates	1.53 ± 2.10 a	4.93 ± 9.54 a	1.71 ± 1.38 a	3.17 ± 1.78 a	x

* Means ± standard deviation in a column followed by the same letter does not differ significantly at α = 0.05 (Duncan test). N = 18 vessels in each treaatment at the beginning of the experiment; N differs from 14 to 17 at the date of evaluation, because of removing of contaminated cultures.

**Table 6:** Effects of type of medium and plant growth regulators (PGRs) and the after effect of different carbohydrates on fresh weight (g) of mature bulbs of the tulip cultivar ‘Heart of Warsaw’ formed in vitro.

Type of Medium, PGRs Added to the Medium	Carbohydrate				Mean for Type of Medium
	Sucrose	Glucose	Fructose	Glucose + Fructose	
1st control, single-phase medium (without liquid medium)	-	0.575 ± 0.34 a *	0.329 ± 0.14 a	0.293 ± 0.06 a	0.412 ± 0.24 a
2nd control, two-phase medium (liquid medium without PGRs)	0.424 ± 0.06 a	0.912 ± 0.43 a	0.748 ± 0.03 a	0.261 ± 0.16 a	0.512 ± 0.31 a
Two-phase medium (liquid medium with 2 mg·L^−1^ NAA)	0.483 ± 0.25 a	0.509 ± 0.02 a	0.732 ± 0.83 a	0.510 ± 0.31 a	0.571 ± 0.44 a
Two-phase medium (liquid medium with 1 mg·L^−1^ PBZ)	0.371 ± 0.30 a	0.265 ± 0.18 a	0.376 ± 0.25 a	0.728 ± 0.27 a	0.470 ± 0.30 a
Mean for carbohydrates	0.419 ± 0.20 a	0.536 ± 0.33 a	0.515 ± 0.43 a	0.486 ± 0.30 a	x

* Means ± standard deviation in a column followed by the same letter does not differ significantly at α = 0.05 (Duncan test). N = 18 vessels in each treatment at the beginning of the experiment; N differs from 14 to 17 at the date of evaluation, because of removing of contaminated cultures.

**Table 7:** Modifications of the MS medium used in the experiment.

Treatment	PGRs Added
1	TDZ 0.1 mg·L^−1^ + NAA 0.1 mg·L^−1^ + 2iP 5 mg·L^−1^
2	TDZ 0.1 mg·L^−1^ + IBA 0.1 mg·L^−1^ + 2iP 5 mg·L^−1^
3	mT 0.1 mg·L^−1^ + NAA 0.1 mg·L^−1^ + 2iP 5 mg·L^−1^
4	mT 0.1 mg·L^−1^ + IBA 0.1 mg·L^−1^ + 2iP 5 mg·L^−1^
5	2iP 0.1 mg·L^−1^ + NAA 0.1 mg·L^−1^ + mT 5 mg·L^−1^
6	2iP 0.1 mg·L^−1^ + IBA 0.1 mg·L^−1^ + mT 5 mg·L^−1^

**Table 8:** Modifications regarding the type and content of carbohydrates in MS medium used in the Experiment 2.

Treatment	Carbohydrate
1	sucrose 30 g·L^−1^
2	glucose 30 g·L^−1^
3	fructose 30 g·L^−1^
4	glucose 15 g·L^−1^ + fructose 15 g·L^−1^

## Data Availability

Data sharing not applicable.

## Supplementary Materials

The following supporting information can be downloaded at: https://www.mdpi.com/article/10.3390/plants12051134/s1.
