# Supplementary figures and images for: The Influence of Selected Plant Growth Regulators and Carbohydrates on In Vitro Shoot Multiplication and Bulbing of the Tulip (Tulipa L.)
Source: Plants (Basel). 2023 Mar 2;12(5):1134. doi: 10.3390/plants12051134 (PMC10005141; doi:10.3390/plants12051134)

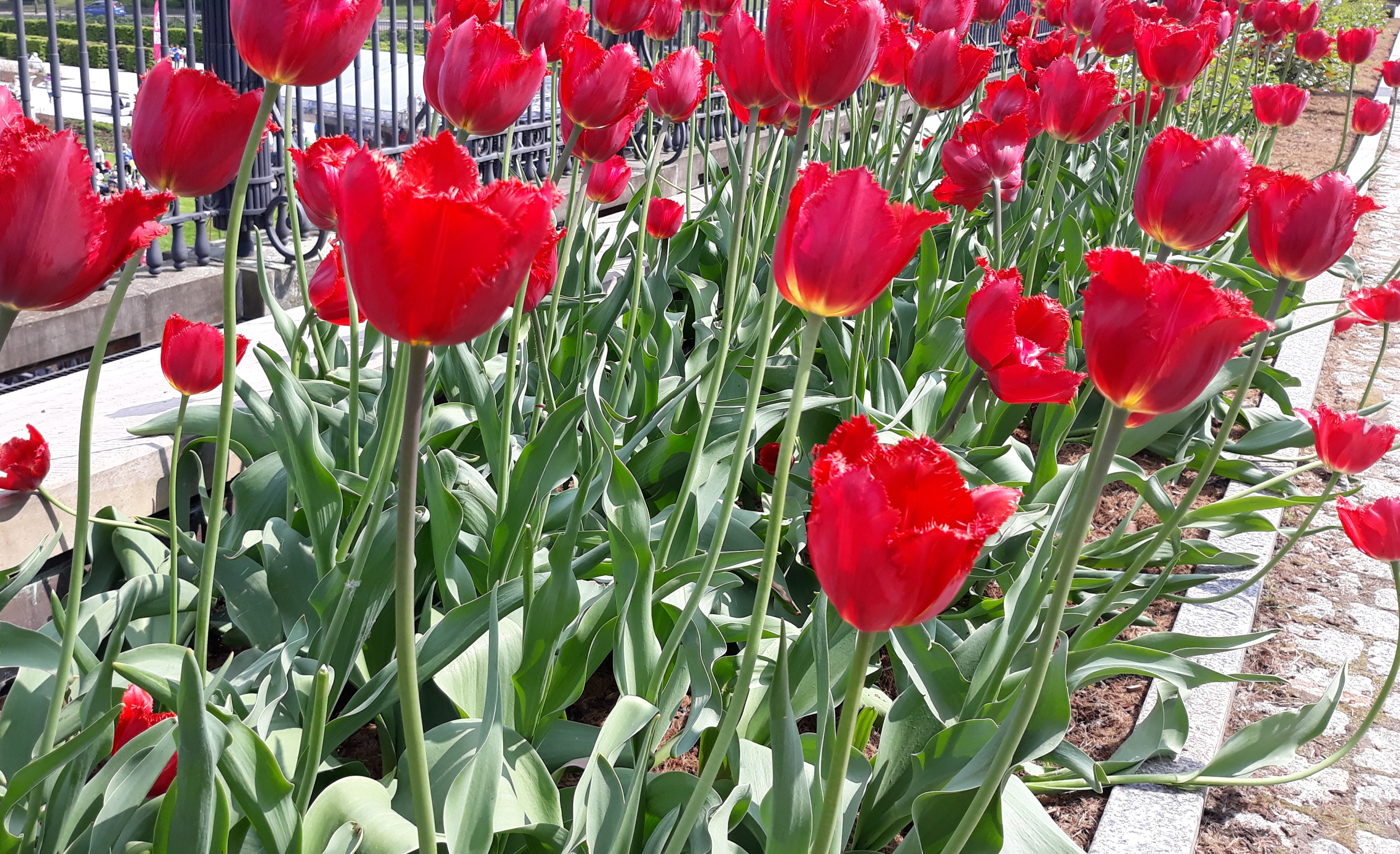

Supplement: Supplementary file 1 [file plants-12-01134-s001.zip › plants-2161108-supplementary.jpg]
